# Machine learning to identify chronic cough from administrative claims data

**DOI:** 10.1038/s41598-024-51522-9

**Published:** 2024-01-30

**Authors:** Vishal Bali, Vladimir Turzhitsky, Jonathan Schelfhout, Misti Paudel, Erin Hulbert, Jesse Peterson-Brandt, Jeffrey Hertzberg, Neal R. Kelly, Raja H. Patel

**Affiliations:** 1 Center for Observational and Real-World Evidence (CORE), Merck & Co, Rahway, NJ USA; 2 Health Economics and Outcomes Research (HEOR), Optum Insight, Eden Prairie, MN USA; 3 OptumLabs, Minnetonka, MN USA

**Keywords:** Machine learning, Respiratory tract diseases

## Abstract

Accurate identification of patient populations is an essential component of clinical research, especially for medical conditions such as chronic cough that are inconsistently defined and diagnosed. We aimed to develop and compare machine learning models to identify chronic cough from medical and pharmacy claims data. In this retrospective observational study, we compared 3 machine learning algorithms based on XG Boost, logistic regression, and neural network approaches using a large claims and electronic health record database. Of the 327,423 patients who met the study criteria, 4,818 had chronic cough based on linked claims–electronic health record data. The XG Boost model showed the best performance, achieving a Receiver-Operator Characteristic Area Under the Curve (ROC-AUC) of 0.916. We selected a cutoff that favors a high positive predictive value (PPV) to minimize false positives, resulting in a sensitivity, specificity, PPV, and negative predictive value of 18.0%, 99.6%, 38.7%, and 98.8%, respectively on the held-out testing set (n = 82,262). Logistic regression and neural network models achieved slightly lower ROC-AUCs of 0.907 and 0.838, respectively. The XG Boost and logistic regression models maintained their robust performance in subgroups of individuals with higher rates of chronic cough. Machine learning algorithms are one way of identifying conditions that are not coded in medical records, and can help identify individuals with chronic cough from claims data with a high degree of classification value.

## Introduction

Administrative data, including insurance claim codes, are useful sources of de-identified patient information that can be incorporated into retrospective clinical research studies^1^. Machine learning and other algorithmic approaches enable the analysis of large volumes of such data in predicting and classifying disease state, modeling disease progression, recommending medical interventions, and predicting future risks^2–7^.

Another powerful use of machine learning is to aid in the identification of populations with, or at risk of clinical conditions, sometimes called phenotyping. More accurate patient identification can accelerate research on the prevalence, characteristics, epidemiology, and burden of such conditions^8–11^. One population that would benefit from improved characterization comprises individuals with chronic cough, currently defined as daily cough for 8 weeks or more^12,13^. Many cases of chronic cough resolve upon successful treatment of common underlying conditions such as asthma, upper airway cough syndrome (formerly postnasal drip), and gastroesophageal reflux disease or cessation of prescription medicines (e.g., angiotensin-converting enzyme [ACE] inhibitors) that can cause cough^14,15^. However, a cause and/or successful treatment cannot be identified for up to half of individuals with chronic cough^12,16,17^. Chronic cough is associated with high rates of health care resource use, and individuals with unexplained or treatment-resistant chronic cough often see multiple specialists and undergo extensive diagnostic testing^18–24^. Patients generally report poor success rates with prescription drugs and other medical approaches; over-the-counter or home remedies for cough are commonly used instead of or in addition to prescription options^21,22,25^. Many individuals report giving up on seeking further medical attention for their chronic cough due to lack of previous success^21,22^.

There is no FDA-approved treatment that is specific to chronic cough, which has generally been regarded as a symptom rather than a distinct clinical entity. In addition, at the time of this study there was also no diagnosis code for chronic cough. Many patients receive other diagnoses before their chronic cough is properly addressed, which impedes their treatment as well as research efforts to characterize and address the unmet diagnostic and therapeutic needs of this population^26,27^.

We and others have recently reported the development and validation of natural language processing (NLP) algorithms that can identify cough mentions from provider notes in patients’ electronic health records (EHRs)^18,27,28^. Our NLP-based algorithm had a positive predictive value (PPV) of 0.96 for identification of cough mentions, as compared to a manually annotated gold standard data set^18^. This algorithm defines chronic cough as the presence of at least 3 cough encounters within a 120-day period, with at least 56 days between the first and last encounters. The 120-days period as a maximum “gap” increases the likelihood that the 3 cough encounters shared a common etiology. This rule-based algorithm heavily relies on the presence of clinical notes. Without clinical notes, the algorithm identified just 15.9% of chronic cough cases^18^. Additionally, clinical notes are only available in smaller EHR databases due to costs of de-identifying such data and privacy concerns. In contrast, many administrative claims database cover large proportions of the population. The objective of the current study was to augment this previous work by developing machine learning algorithms to identify and characterize individuals with chronic cough from medical and pharmacy claims data.

## Results

### Participants

**Figure 1:**
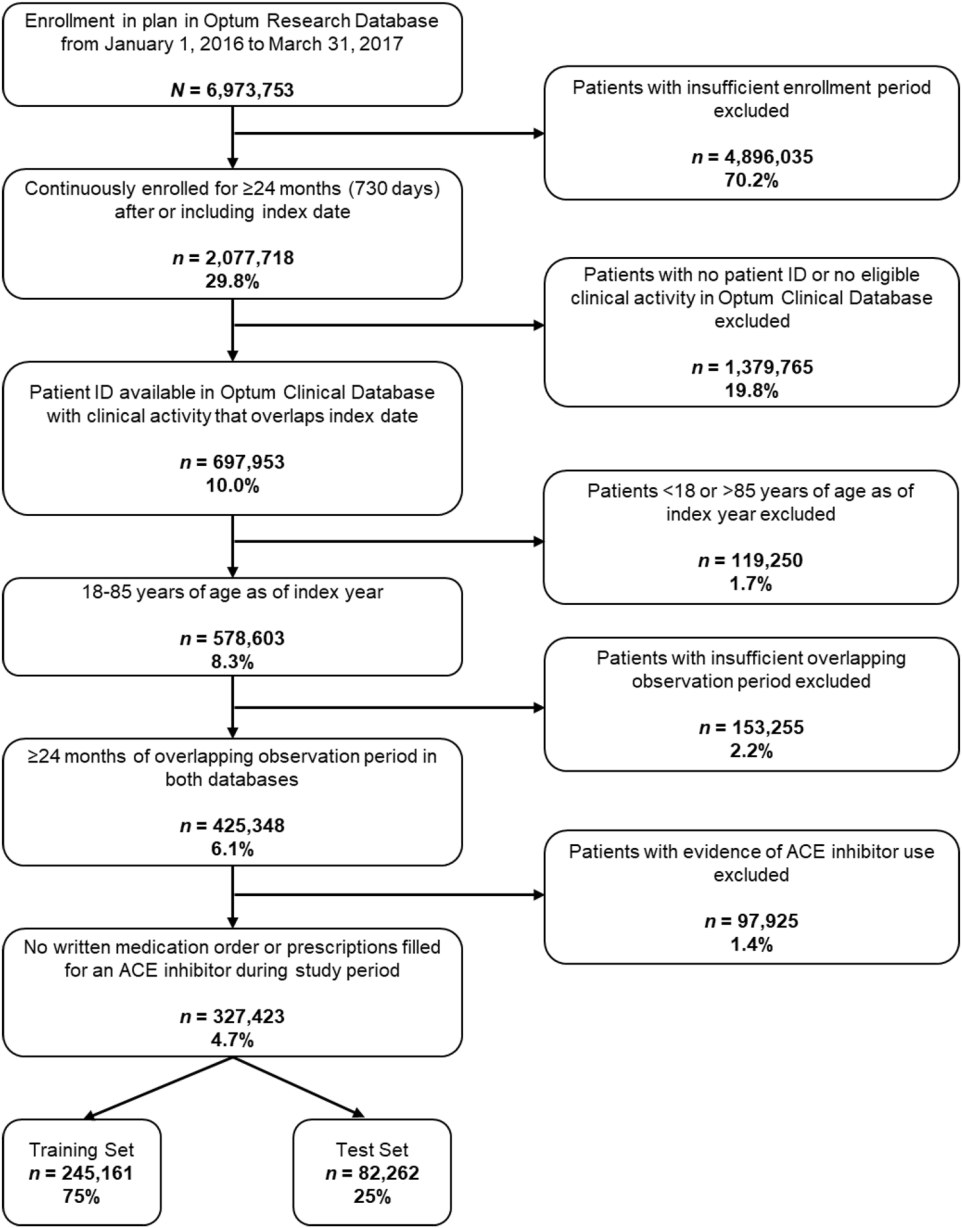
Cohort attrition diagram.

The final sample was identical to that reported previously and comprised 327,423 individuals 18–85 years of age with at least 24 months of claims data and no evidence of ACE inhibitor use (Fig. 1)^18^. The gold standard EHR-based algorithm identified a total of 128,467 individuals with ≥ 1 cough encounter and 4,818 (1.5%) who met the criteria for chronic cough^18^. Among the gold standard positive class for chronic cough, 66.7% were female and the mean (SD) age was 61.0 (15.3)^18^.

### Performance of 3 claims-based chronic cough identification models

Performance metrics for 3 claims-based identification models for chronic cough (XG Boost, logistic regression, and neural network approaches) are summarized in Table 1. All 3 models had high specificity (0.974–0.996) and negative predictive value (NPV; 0.985–0.988). PPV (also termed precision) was 0.134 for the XG Boost-based model, 0.344 for the logistic regression model, and 0.218 for the neural network-based model. Sensitivity (recall) was low (0.153–0.207) across all 3 models.

**Table 1:** Performance of 3 claims-based predictive models of chronic cough.

Metric	XG Boost	Logistic regression	Neural network
Sensitivity (recall)	0.207	0.153	0.201
PPV (precision)	0.134	0.344	0.218
Specificity	0.974	0.996	0.989
NPV	0.985	0.987	0.988
Accuracy	0.960	0.983	0.977
ROC-AUC	0.916	0.907	0.838
PR-AUC	0.229	0.205	0.135

*NPV* negative predictive value; *PPV* positive predictive value; *PR-AUC* precision-recall, area under the curve; *ROC-AUC* receiver-operating characteristic, area under the curve.

**Figure 2:**
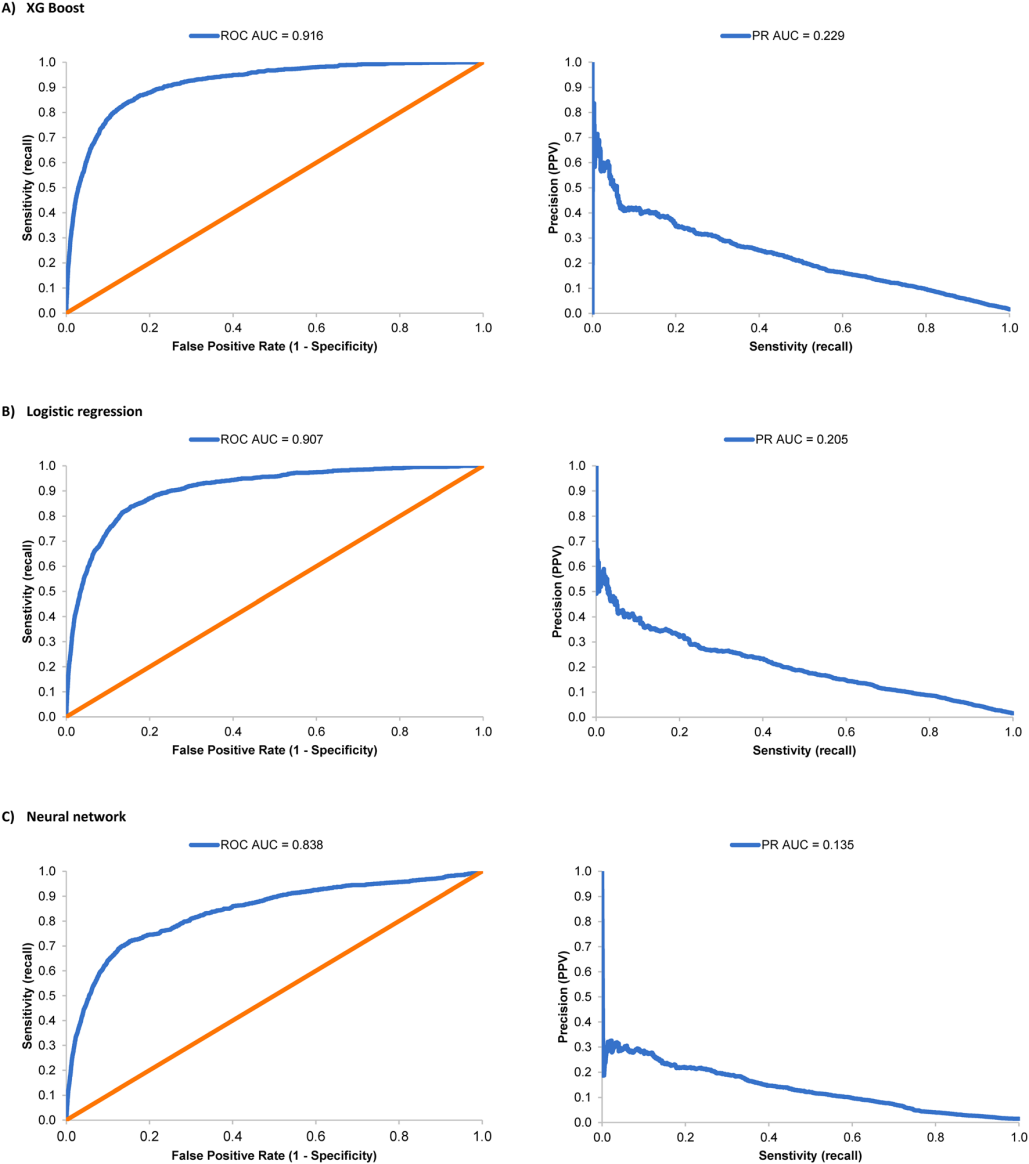
Comparative performance of 3 identification models of chronic cough. Receiver-operating characteristic (left) and precision-recall (right) curves are shown for 3 classification models of chronic cough, based on XG Boost (**A**), logistic regression (**B**), and neural networks (**C**). PPV, positive predictive value; ROC-AUC, receiver-operating characteristic, area under the curve. The orange line on each ROC plot represents the performance of a hypothetical random model with an AUC of 0.5.

Receiver-operating characteristic (ROC) and precision-recall (PR) curves were plotted for each model (Fig. 2). The former plots represent the tradeoff between sensitivity and specificity, while the latter visualize the tradeoff between sensitivity rate and PPV. The XG Boost-based model produced the highest area under the curve (AUC) values for both ROC and PR, while the neural network-based model had the lowest values for both metrics.

Feature importance for the first 50 variables for the XGBoost model and the logistic model have been reported in supplementary Tables. The most important identification features for the XG Boost-based model were having a chest X-ray (Current Procedural Terminology [CPT] code 71,020), count of cough diagnoses (ICD-10 R05), a prescription for albuterol, a visit with a pulmonary specialist, and an office visit (CPT 99,215). For the logistic regression model, the most important identification features were ≥ 1 cough symptom (ICD-10 R05; coefficient = 1.012), ≥ 3 cough diagnoses (ICD-10 R05; coefficient = 0.820), a pulmonary specialist encounter (coefficient = 0.521), ≥ 3 home visits for nursing care (Healthcare Common Procedure Coding System code S9123; coefficient = 0.465), and residing in the Midwest region of the US (Illinois, Indiana, Iowa, Kansas, Michigan, Minnesota, Missouri, Nebraska, North Dakota, Ohio, South Dakota, or Wisconsin; coefficient = –0.461).

Chronic cough is consistently reported to be more common in women than in men, and to predominantly affect individuals > 50–60 years of age^18,19,29–31^. We therefore also assessed the performance of the 3 models with 3 subpopulations found to have a higher prevalence of chronic cough; females (chronic cough prevalence of 1.65% in our sample), individuals ≥ 65 years of age (prevalence 2.77%), and individuals with a diagnosis of cough (prevalence 5.39%). The most favorable performance metrics were produced by the logistic regression model for individuals diagnosed with cough, and by the XG Boost model for females and those ≥ 65 years of age (Table 2).

**Table 2:** Performance of predictive models of chronic cough in subpopulations with a higher prevalence of chronic cough.

	Full sample	Individuals with cough diagnoses	Females	Individuals ≥ 65 years of age
ROC-AUC				
XG Boost	0.916	0.817	0.914	0.898
Logistic regression	0.907	0.823	0.902	0.882
Neural network	0.838	0.684	0.837	0.829
PR-AUC				
XG Boost	0.229	0.103	0.225	0.288
Logistic regression	0.205	0.241	0.207	0.244
Neural network	0.135	0.055	0.138	0.167

*PR-AUC* precision-recall, area under the curve; *ROC-AUC* receiver-operating characteristic, area under the curve.

## Discussion

In this study we developed and compared 3 claims-based identification models for prevalent chronic cough, based on XG Boost, logistic regression, and neural network machine learning approaches. The rationale was to explore 3 different models with varying range of complexity/flexibility to help understand variation in their performance. Logistic regression is a simple model with good interpretability. On the other hand, neural network models offer the most flexibility with the least interpretability. XGBoost is a tree-based model like Random Forests and offers a compromise between interpretability and flexibility. These 3 models were selected because they are commonly used models which most data scientists are familiar with. All models had high specificity and NPV indicating that individuals without chronic cough were identified well. Comparatively lower PPV (precision) and sensitivity (recall) were observed due to a very small proportion of the positive class in the sample population (1.5% observed to have chronic cough). Considering this imbalance, logistic regression model was able to achieve a 34% PPV from the full study sample of medical plan enrollees. The most common metric used to measure classification performance is ROC-AUC, using which XG Boost had the best performance, closely followed by logistic regression model.

We also applied each model to 3 subsamples with a higher prevalence of chronic cough compared to the overall sample. The relative performance of the 3 models differed across these subsamples, with the logistic regression model having the most favorable ROC-AUC and PR-AUC metrics of the 3 when applied to individuals diagnosed with cough, and the XG Boost-based model performing best in the female and ≥ 65 years of age subsamples.

The 3 models had distinct but overlapping sets of most important features for chronic cough identification. Seeing a pulmonary specialist and having a higher number of cough diagnoses were important features in both the XG Boost and the logistic regression models. One of the drawbacks of neural network models is that they are a “black box” method and do not enable identification of the variables driving the algorithm; research to develop reporting methods to address this issue is ongoing^32^.

To our knowledge, this is the first study to use machine learning approaches to identify individuals with chronic cough based solely on medical and pharmacy claims data. The study sample had an estimated chronic cough prevalence of 1.5%, which is lower than recent estimates of ~ 5% from general population surveys in both the UK and US (although similar to an estimate of 1.04% from our previous work that developed an NLP algorithm for chronic cough and deployed it in a different sample population)^19,23,27^. We hypothesize that the NLP rule-based algorithm that formed the chronic cough population in this study missed many individuals with the condition, as seen by the low prevalence compared to previous population prevalence estimates; though evaluating the sensitivity of the rule-based algorithm, either with chart review or surveys, was not performed in this work.

We had expected that the most prominent features in our models would relate to known risk factors for chronic cough such as smoking, bronchitis, asthma, gastroesophageal reflux disease, and COPD. Some of these conditions were selected in our models and were included among the top 50 most prominent features, but they were not consistently within the top 10 most important features. For example, a diagnosis of ‘other COPD’ was the seventh most important feature in the XG Boost-based model, while ‘acute bronchitis’ ranked seventeenth. Smoking was not a prominent feature for the XG Boost-based and logistic regression models. However, diagnosis and other codes for smoking or smoking cessation are generally not well populated in claims data^33^. Incentives for US providers to record smoking-related codes were introduced in 2010 as part of the ‘meaningful use of certified electronic health record’ policy, and have been reported to increase the sensitivity of claims-based approaches to identifying smoking status^34^. Smoking might therefore become a more important feature in future iterations of machine learning models to predict chronic cough and other smoking-related conditions. In general, it is likely that the most important predictive features of models designed to detect individuals at high risk of developing chronic cough would differ from those observed in our models, which were designed to identify individuals with current chronic cough.

A strength of the current study is that the databases we used to train, validate, and test our models included full medical and pharmacy claims data for individuals representing ~ 19% of the US commercially insured population, ~ 21% of the Medicare Advantage population, and ~ 22% of the Medicare Part D population. As such, our sample is more nationally representative of the US population than any other single provider system sample. Since different clinical claims databases contain different data types, our comparative analysis of the most prominent features for 3 machine learning models could aid in the development of guidelines for optimal model selection customized to the characteristics of individual data sets. Similarly, our comparative subsample analysis could help to guide optimal model selection for populations with different chronic cough risk profiles.

The neural network model achieved the least favorable performance of the 3 algorithms in identifying individuals with chronic cough in the study population and subgroups. This may be due to the small sample size and the relatively limited information in structured claims data. Deep learning models may be more advantageous when the source data includes free-form text or images, such as clinical notes or radiology images.

One inherent limitation of this study was the under-coding of cough diagnoses in the structured claims data.. Future studies will aim to examine and validate the performance of the machine learning models developed in this study when incorporating the new ICD-10-CM code of R05.3 for chronic cough^35^. Another limitation is that our data set included records from a network of > 140,000 providers and thus may include significant heterogeneity in how cough diagnoses and claims are recorded. In addition, medical claims data are subject to coding errors^1^. Further, diagnosis codes do not always indicate definite disease presence and in some cases may be used to rule diagnoses out, while prescription claims or written orders do not necessarily indicate that a medication was taken as prescribed. Also, given the low prevalence of the positive class, we could not stratify and test how well the model performed across different geographic locations.

## Conclusions

Our findings can be used in payer and provider systems to identify individuals with chronic cough who may benefit from further diagnostic testing and treatment, and to identify representative populations of individuals with chronic cough to aid in clinical research. However, more work is needed to improve the PPV and sensitivity of identification models for chronic cough. Overall, we suggest that logistic regression be prioritized for future model development work on administrative claims data, due to its robust performance in the overall sample and in subsamples of individuals at higher risk for chronic cough, as well as its ease of use. Additionally, further work needs to be done to better understand use of different machine learning models in identification of chronic cough patients.

## Methods

### Study design

This was a retrospective observational study. All data and databases used in this study were statistically deidentified, and all study procedures were compliant with the United States Health Insurance Portability and Accountability Act. The study therefore did not require Institutional Review Board approval or informed consent.

### Study sample

The sample population was drawn from Optum’s Integrated Clinical + Claims Database, which combines adjudicated medical and pharmacy claims with EHRs from the Optum Research Clinical Database. The latter database currently has > 101 million unique patients from ~ 60 provider delivery organizations in the United States and Puerto Rico, with an average of 45 months of observed data per patient. The integrated database includes health plan enrollment data; clinical information, including medications prescribed and administered; lab results, vital signs, and body measurements; diagnoses and procedures; and information derived from provider notes using proprietary NLP methods.

The data used in this study were from 01 January 2016 through 31 March 2019. Inclusion and exclusion criteria were as described in Bali et al. ^18^. Briefly, eligible participants were enrolled in a national commercial or Medicare Advantage medical and pharmacy plan in the Optum Research Clinical Database between January 2016 and March 2017, with their earliest enrollment date set as the index date. Continuous enrollment for at least 24 months after and including the index date was required for inclusion in the data set, and eligible participants were 18–85 years of age as of their index year. Enrollees were excluded from the study if their EHRs included evidence of a pharmacy fill or written medication order for an ACE inhibitor, a class of blood pressure medication that can cause chronic cough. Eligible participants were divided into training/validation (75%, *n* = 245,161) and test (25%, *n* = 82,262) data sets. The number of positive sample class (i.e., with chronic cough) were 3,651 (1.49%) and 1,167 (1.42%) in the training and test set, respectively. The number of negative sample class (i.e., without chronic cough) were 241,510 (98.5%) and 81,095 (98.6%) in the training and test set, respectively.

### Development of 3 claims-based chronic cough identification models

The gold standard positive class for the development of the claims-based algorithm was generated by implementing an NLP algorithm we developed previously using data available from EHRs^28^. This algorithm has also been replicated using the Kaiser Permanente Southern California Research Data Warehouse using the same integrated database as in the current study^27^. The algorithm defines chronic cough as the presence of at least 3 cough encounters (any combination of 3 sources of information: NLP-identified mentions of ‘tussis’ or any inflection of the word ‘cough’ in free-text provider notes, occurrences of acute cough-specific diagnosis code ICD-10 R05, or written medication orders for benzonatate or dextromethorphan) in EHRs within a 120-day period, with at least 56 days between the first and last cough encounters. The negative class comprised all individuals not identified as part of the gold standard positive class.

Three claims-based classification models were constructed using supervised machine learning methods to identify individuals with chronic cough from the sample population. The models were based on Extreme Gradient Boosted Trees (XG Boost, a decision tree ensemble), logistic regression, and neural network ensemble approaches; see below for methodological details specific to each model.

All models were constructed using diagnosis, procedure, prescription, provider specialty, and patient demographic information from the individuals in the sample. Diagnosis, procedure, provider specialty, and prescription features consisted of count values for each patient to represent the total number of occurrences (i.e., doctor visits, prescriptions filled, diagnoses received, etc.) of the same code the patient has received over the 24-month observation period. Demographic (age, regional location, & gender) and insurance type information was represented as indicators. In addition, the International Statistical Classification of Diseases and Related Health Problems Procedural Classification System (ICD-PCS) was used to map all procedures to higher-level (3-digit) procedure groupings using Clinical Classification Software developed by the Healthcare Cost and Utilization Project^36^. Generic Product Identifier groupings were used for National Drug Codes; we used the first 8 digits of the Generic Product Identifier to determine the generic drug name.

Feature selection was undertaken to reduce the number of independent variables used to identify chronic cough by removing irrelevant or less relevant features that negatively affected model performance. Feature importance was based on information gain for the XGBoost model and output of the LASSO procedure for the logistic model. Features were created using data from the index date (first chronic cough date for positive class or earliest enrollment date for negative class) through the next 24 months. A minimum code prevalence cutoff of 0.04 was used for all models. Since the data set comprised predominantly categorical variables, relevant classification features of the classifier were identified by setting odds ratio thresholds where the confidence intervals were < 0.8 or > 1.2. Any features not associated with chronic cough would have odds ratios close to 1.0 and would be effectively removed^37^. Implementing this process across all 3 machine learning models reduced the overall number of independent features by 90%. Additional feature selection processes that were unique to each model were also used; details are provided below.

Nested three-fold cross-validation (resampling to determine model performance on a held-out data set) was performed to select features and hyperparameters for all models^38^. Model performance during cross-validation was based on average precision (PR-AUC). Final model coefficients and hyperparameters were fit on the entire training set and performance was evaluated on the test set (25% of the data). We also assessed the performance of the 3 models with subpopulations from the test set comprising individuals with a diagnosis of cough, females, and individuals ≥ 65 years of age.

Different binary classification methods were developed and applied to determine the best algorithm to appropriately identify chronic cough. All 3 models incorporated cross-validation and hyperparameter tuning to optimize performance. For all models, a probability threshold of 0.5 was initially used to define accurate classification of chronic cough, and then adjusted to maximize precision and specificity. The final models used the following probability thresholds: XG Boost, 0.70; logistic regression, 0.30; neural network, 0.85. Secondary analyses stratified and evaluated model performance in the test data set among individuals with cough diagnoses (defined as any instance of an ICD-10 R05 code in the individual’s history), female participants, and participants  ≥ 65 years of age. A probability threshold of 0.70 was used for the XG Boost model in the cough diagnosis subgroup due to the small sample size.

#### XG Boost-based model

XGBoost is an ensemble-based decision tree algorithm that uses boosting and gradient descent to assign and adjust the weights on each tree, minimizing loss^39^. Ensemble methods fit an initial tree-based algorithm to the data, then build a second version of the model to improve upon (boost) the classification of the first. The process is iterated until the classification error (mean squared error) no longer decreases with each subsequent boost (i.e., gradient descent).

A Bayesian search method was used in tandem with a threefold cross-validation to tune the model’s hyperparameters, in order to modify the constraints in constructing each tree during cross-validation. While optimizing for average precision we limited the maximum depth of the tree, the minimal weight needed for each child node, and the minimum loss reduction needed to progress further in a leaf node; we also tuned the balance between positive and negative weights and the subsampling ratio for each tree and training instance^40^. The final optimized hyperparameter values selected after tuning and cross-validation are listed in Supplemental Table 1. Diagnosis, procedure, provider specialty, and prescription features were represented as count values for each patient, corresponding to the total number of occurrences with the same code for each patient during the study period. Demographic data (age, regional location, binary gender) and insurance type information were represented as indicators.

#### Logistic regression model

For the logistic regression model, all features were converted to flags, so that counts became flags for 1 or more, 2 or more, and 3 or more. We constructed a logistic regression model with L1 regularization (i.e., least absolute shrinkage and selection operator [Lasso] regression)^41^. This technique adds a regularization term to the equation as a hyperparameter that acts as a penalty term to avoid overfitting. As a result, the most important features within the model may be assigned higher final coefficients, while less important features are ultimately set to zero. This is a particular strength in a data set with sparse data or a large number of features. In general, a hyperparameter that is too small will result in no regularization term and essentially an unpenalized logistic regression model. In such cases, the model will be overfit. Conversely, a larger hyperparameter will add too much weight and can lead to an underfit model.

Grid search was applied to the model along with a K-fold cross-validation method to tune for class weight and regularization strength (C) while optimizing for average precision. As the model was fit on the training set and internally validated on a held-out validation set, the K-fold cross-validation process (K = 3) was performed 3 times with a different randomly selected subsample held-out each time. Different values were tested for the C parameter (0.1, 1, and 10); the optimized value was 0.1. The class weight parameter was calibrated between a balanced weighting and its default weight assignment; the tuned model was optimized to use its default method where both positive and negative classes had equal weighting within the cost function of the algorithm^42^. All independent variables passed into the logistic regression model were represented as binary indicators of 0 or 1. Each diagnosis or procedure code was assigned one of 3 categories based on how frequently the code occurred for each patient (≥ 1, ≥ 2, or ≥ 3).

#### Neural network (deep learning)-based model

Neural networks, also known as deep learning models, comprise a series of algorithms developed to recognize patterns in data that identify an outcome—in this case, presence of chronic cough. Neural networks mimic the function of the human brain via layers of interconnected neurons. Simple neural networks comprise input, hidden, and output layers. We developed a neural network using Tensorflow software^43^. Hyperparameters were tuned using KerasTuner^44^. The model consisted of a multi-layered neural network, in which each layer contained 100 neurons and used the scaled exponential linear unit activation function^45^. The output layer used a sigmoid function to produce a probability that conveyed the likelihood of a given patient being classified as positive for chronic cough. The model used an L2 penalty as a regularization technique, which adds a penalty to the model that is equal to the square of the magnitude of the coefficients, to avoid overfitting^46^. Unlike L1/Lasso methods, L2 regularization does not drive coefficients down to zero and therefore does not reduce data dimensionality. We used LAMB as the optimizer and binary cross entropy as the loss function^47,48^.

Using the KerasTuner framework to hyperparameter tune the neural network, we implemented a Bayesian optimization method to tune the number of hidden layers in the model, the dropout rate, and the magnitude of the L2 regularization penalty to create a model optimized for minimal loss. During the hyperparameter optimization process we tuned the L2 penalty rate at 0.01, 0.001, and 0.0001; the optimized value was chosen to be 0.0001. We also tuned the overall length of the neural network to have 1–5 hidden layers, with each layer separately tuned to a dropout rate between 0 and 0.3; the optimized model was tuned to have only one hidden layer with a dropout rate of 0.3 (the optimized hyperparameters and additional dropout rates for more hidden layers are listed in Supplemental Table 1).

### Statistical analysis

All study variables were analyzed descriptively. Means and SDs were calculated for continuous variables. Statistical analyses were performed using SAS software (SAS Institute, Cary, NC) and/or R, Python, and Spark.

### Ethical approval and consent to participate

All data and databases were accessed in compliance with US patient confidentiality requirements, including the Health Insurance Portability and Accountability Act of 1996 regulations (HIPAA). All databases used are de-identified and fall under the US Department of Health and Human Services Policy for the Protection of Human Subjects, and therefore informed consent and Institutional Review Board approval were not required.

### Supplementary Information

Supplementary Information.

## Data Availability

The data that support the findings of this study are available from Optum’s Integrated Clinical + Claims Database but restrictions apply to the availability of these data, which were under license for the current study, and so are not publicly available. Data are however available from the corresponding author upon reasonable request and with permission of Optum.

## Abbreviations

ACE: Angiotensin-converting enzyme
AUC: Area under the curve
CPT: Current procedural terminology
EHR: Electronic health record
ICD-PCS: International statistical classification of diseases and related health problems procedural classification system
NLP: Natural language processing
NPV: Negative predictive value
PPV: Positive predictive value
PR: Precision-recall
ROC: Receiver-operating characteristic
