# Supplementary material for: Machine learning to identify chronic cough from administrative claims data
Source: Sci Rep. 2024 Jan 30;14:2449. doi: 10.1038/s41598-024-51522-9 (PMC10828499; doi:10.1038/s41598-024-51522-9)
Supplement: Supplementary file 1 — Supplementary Information. [file 41598_2024_51522_MOESM1_ESM.docx]

**Supplementary Materials**

**Supplemental Table 1. Optimized hyperparameter values for XG Boost-based and neural network-based predictive models for chronic cough**

| **Hyperparameter** | **Description** | **Default value and/or (range)** | **Optimized value** |
| --- | --- | --- | --- |
| **XG Boost-based predictive model** | | | |
| n_estimators | Total number of trees in an XGBoost model. | 100 | 400 |
| learning_rate | Determines the contribution of each tree to the final outcome, and how quickly the algorithm proceeds down the gradient descent.  Smaller values result in a model that is more robust to characteristics and avoids overfitting. | (0–1) | 0.02 |
| max_depth | Represents the maximum number of layers and controls the size of the decision trees.  Deeper trees (larger max_depth values) run a high risk of overfitting. | 3 | 4 |
| subsample | Subsampling ratio of training instances; used to prevent overfitting.  A value of 0.5 would mean that XGBoost randomly samples half the training data prior to expanding trees during boosting iterations. | 1  (0–1) | 0.8 |
| colsample_bytree | Subsample ratio of columns when constructing each tree. | 1 | 0.7 |
| min_child_weight | Represents when any further partitioning of a node should stop and sets a minimum value for the sum of all weights in branches below this node.  Larger values result in more conservative models. | 1 | 4 |
| gamma | Minimum loss reduction required to make any further splits on a leaf node of a tree.  Larger values result in more conservative algorithms. | 0 | 0.50 |
| scale_pos_weight | Controls the balance of positive and negative samples.  Value would be 1 if using an equal number of positive and negative class patients. | NA | 33.7 |
| **Neural network-based predictive model** | | | |
| l2_penalty | L2 penalty applied to the model. Values of zero would result in no penalty, and higher values would add more weight. Typical values range from 0 to 0.1 |  | 0.0001 |
| num_layers | The number of hidden layers in the neural network. A neural network with no hidden layers would be equivalent to logistic regression |  | 1 |
| drop_rate_0 | The dropout rate for the first layer |  | 0.3 |
| drop_rate_1 | The dropout rate for the second layer |  | 0.1 |
| drop_rate_2 | The dropout rate for the third layer |  | 0.3 |
| drop_rate_3 | The dropout rate for the fourth layer |  | 0.2 |
| drop_rate_4 | The dropout rate for the fifth layer |  | 0 |

NA, not applicable

**Supplemental Table 2: Top 50 important features from XGBoost Model for Identification of chronic cough**

| **Rank** | **Code Type** | **Value** | **Description** |
| --- | --- | --- | --- |
| 1 | CPT | 71020 | The total number of occurrences a patient has received the procedure: ‘Radiologic examination, chest, 2 views, frontal and lateral’. |
| 2 | ICD10 Category | R05 | The total number of occurrences a patient has received the diagnosis: ‘COUGH’. |
| 3 | GPI8 | 44201010 | The total number of times a patient was prescribed ALBUTEROL. |
| 4 | Provider Specialty | 42 | The total number of times a patient had visited a Pulmonary specialist. |
| 5 | CPT | 99214 | The total number of occurrences a patient has received the procedure: ‘Office or other outpatient visit for the evaluation and management of an established patient, which requires at least 2 of these 3 key components: A detailed history; A detailed examination; Medical decision making of moderate complexity. Counseling and/or coordination of care with other physicians, other qualified health care professionals, or agencies are provided consistent with the nature of the problem(s) and the patient's and/or family's needs. Usually, the presenting problem(s) are of moderate to high severity. Typically, 25 minutes are spent face-to-face with the patient and/or family.’ |
| 6 | CPT | 71046 | The total number of occurrences a patient has received the procedure: ‘Radiologic examination, chest; 2 views’. |
| 7 | ICD10 Category | J44 | The total number of occurrences a patient has received the diagnosis: ‘OTHER CHRONIC OBSTRUCTIVE PULMONARY DISEASE’. |
| 8 | CPT | 94729 | The total number of occurrences a patient has received the procedure: ‘Diffusing capacity (eg, carbon monoxide, membrane)’. |
| 9 | GPI8 | 22100045 | The total number of times a patient was prescribed PREDNISONE. |
| 10 | GPI8 | 03400010 | The total number of times a patient was prescribed AZITHROMYCIN. |
| 11 | ICD10 Category | R91 | The total number of occurrences a patient has received the diagnosis: ‘ABNORMAL FINDINGS ON DIAGNOSTIC IMAGING OF LUNG’. |
| 12 | ICD10 Category | J01 | The total number of occurrences a patient has received the diagnosis: ‘ACUTE SINUSITIS’. |
| 13 | GPI8 | 44209902 | The total number of times a patient was prescribed IPRATROPIUM-ALBUTEROL. |
| 14 | CPT | 96549 | The total number of occurrences a patient has received the procedure: ‘Unlisted chemotherapy procedure’. |
| 15 | GPI8 | 43102010 | The total number of times a patient was prescribed BENZONATATE. |
| 16 | Provider Specialty | 16 | The total number of times a patient had visited a Diagnostic Radiology specialist. |
| 17 | ICD10 Category | J20 | The total number of occurrences a patient has received the diagnosis: ‘ACUTE BRONCHITIS’. |
| 18 | ICD10 Category | J18 | The total number of occurrences a patient has received the diagnosis: ‘PNEUMONIA UNSPECIFIED ORGANISM’. |
| 19 | CPT | 99233 | The total number of occurrences a patient has received the procedure: ‘Subsequent hospital care, per day, for the evaluation and management of a patient, which requires at least 2 of these 3 key components: A detailed interval history; A detailed examination; Medical decision making of high complexity. Counseling and/or coordination of care with other physicians, other qualified health care professionals, or agencies are provided consistent with the nature of the problem(s) and the patient's and/or family's needs. Usually, the patient is unstable or has developed a significant complication or a significant new problem. Typically, 35 minutes are spent at the bedside and on the patient's hospital floor or unit.’ |
| 20 | ICD10 Category | J98 | The total number of occurrences a patient has received the diagnosis: ‘OTHER RESPIRATORY DISORDERS’. |
| 21 | ICD10 Category | J06 | The total number of occurrences a patient has received the diagnosis: ‘ACUTE UPPER RESP INFECTIONS MX & UNS SITES’. |
| 22 | CPT | 99213 | The total number of occurrences a patient has received the procedure: ‘Office or other outpatient visit for the evaluation and management of an established patient, which requires at least 2 of these 3 key components: An expanded problem focused history; An expanded problem focused examination; Medical decision making of low complexity. Counseling and coordination of care with other physicians, other qualified health care professionals, or agencies are provided consistent with the nature of the problem(s) and the patient's and/or family's needs. Usually, the presenting problem(s) are of low to moderate severity. Typically, 15 minutes are spent face-to-face with the patient and/or family.’ |
| 23 | Patient Age | 65+ | Patient of age 65 or above. |
| 24 | ICD10 Category | J45 | The total number of occurrences a patient has received the diagnosis: ‘ASTHMA’. |
| 25 | CPT | 99232 | The total number of occurrences a patient has received the procedure: ‘Subsequent hospital care, per day, for the evaluation and management of a patient, which requires at least 2 of these 3 key components: An expanded problem focused interval history; An expanded problem focused examination; Medical decision making of moderate complexity. Counseling and/or coordination of care with other physicians, other qualified health care professionals, or agencies are provided consistent with the nature of the problem(s) and the patient's and/or family's needs. Usually, the patient is responding inadequately to therapy or has developed a minor complication. Typically, 25 minutes are spent at the bedside and on the patient's hospital floor or unit.’ |
| 26 | GPI8 | 42200032 | The total number of times a patient was prescribed FLUTICASONE. |
| 27 | GPI8 | 05000034 | The total number of times a patient was prescribed LEVOFLOXACIN. |
| 28 | Insurance Coverage | Commerical/Medicare | An indicator that represents if the patient is covered by both Medicare and a commerical insurance plan. |
| 29 | Patient Location | Midwest | An indicator that representing if the patient's residence is in the midwest region of the US. |
| 30 | CPT | 71250 | The total number of occurrences a patient has received the procedure: ‘Computed tomography, thorax; without contrast material’. |
| 31 | GPI8 | 05000037 | The total number of times a patient was prescribed MOXIFLOXACIN HCL. |
| 32 | ICD10 Category | K21 | The total number of occurrences a patient has received the diagnosis: ‘GASTRO-ESOPHAGEAL REFLUX DISEASE’. |
| 33 | Provider Specialty | 50 | The total number of times a patient had visited a provider within a hospital. |
| 34 | ICD10 Category | B97 | The total number of occurrences a patient has received the diagnosis: ‘VIRAL AGENTS CAUSE DISEASES CLASSIFIED ELSEWHERE’. |
| 35 | Patient Age | 18-39 | Patient between the ages of 18 and 39. |
| 36 | ICD10 Category | J40 | The total number of occurrences a patient has received the diagnosis: ‘BRONCHITIS NOT SPECIFIED AS ACUTE OR CHRONIC’. |
| 37 | CPT | 88142 | The total number of occurrences a patient has received the procedure: ‘Cytopathology, cervical or vaginal (any reporting system), collected in preservative fluid, automated thin layer preparation; manual screening under physician supervision’. |
| 38 | CPT | 99223 | The total number of occurrences a patient has received the procedure: ‘Initial hospital care, per day, for the evaluation and management of a patient, which requires these 3 key components: A comprehensive history; A comprehensive examination; and Medical decision making of high complexity. Counseling and/or coordination of care with other physicians, other qualified health care professionals, or agencies are provided consistent with the nature of the problem(s) and the patient's and/or family's needs. Usually, the problem(s) requiring admission are of high severity. Typically, 70 minutes are spent at the bedside and on the patient's hospital floor or unit.’. |
| 39 | Insurance Coverage | Invalid/Missing/Unknown/Other | An indicator representing if the patient's insurance coverage is not specified. |
| 40 | ICD10 Category | R06 | The total number of occurrences a patient has received the diagnosis: ‘ABNORMALITIES OF BREATHING’. |
| 41 | GPI8 | 02300040 | The total number of times a patient was prescribed CEFDINIR. |
| 42 | GPI8 | 27104006 | The total number of times a patient was prescribed INSULIN DETEMIR. |
| 43 | Patient Location | West | An indicator that representing if the patient's residence is in the west region of the US. |
| 44 | ICD10 Category | C79 | The total number of occurrences a patient has received the diagnosis: ‘SECONDARY MALIGNANT NEOPLASM OF OTHER AND UNSPECIFIED’. |
| 45 | ICD10 Category | R13 | The total number of occurrences a patient has received the diagnosis: ‘APHAGIA AND DYSPHAGIA’. |
| 46 | CPT | 87591 | The total number of occurrences a patient has received the procedure: ‘Infectious agent detection by nucleic acid (DNA or RNA); Neisseria gonorrhoeae, amplified probe technique’. |
| 47 | CPT | 87491 | The total number of occurrences a patient has received the procedure: ‘Infectious agent detection by nucleic acid (DNA or RNA); Chlamydia trachomatis, amplified probe technique’. |
| 48 | GPI8 | 90450030 | The total number of times a patient was prescribed SILVER SULFADIAZINE. |
| 49 | CPT | 71260 | The total number of occurrences a patient has received the procedure: ‘Computed tomography, thorax; with contrast material(s)’. |
| 50 | HCPCS | S9123 | The total number of occurrences a patient has received the procedure: ‘Nursing care, in the home; by registered nurse, per hour’. |

**Supplementary Table 3: Output from the LASSO procedure for the logistic model for Identification of chronic cough**

| **Rank** | **Coefficients** | **Features** | **Feature Descriptions** |
| --- | --- | --- | --- |
| 1 | 1.012 | counts_of_ICD10CM-cat_R05_1_or_more | Patient had 1 or more occurrences of the diagnosis: COUGH. |
| 2 | 0.820 | counts_of_ICD10CM-cat_R05_3_or_more | Patient had 3 or more occurrences of the diagnosis: COUGH. |
| 3 | 0.521 | counts_of_prvspec_42_1_or_more | Patient had visited a Pulmonary specialist 1 or more times. |
| 4 | 0.465 | counts_of_HCPCS_S9123_3_or_more | Patient had 3 or more occurrences of the procedure: Nursing care, in the home; by registered nurse, per hour (use for general nursing care only, not to be used when CPT codes 99500-99602 can be used). |
| 5 | -0.461 | region_MIDWEST | Patients residing in the MIDWEST region. |
| 6 | -0.410 | ins_coverage_uninsured | Patient not covered by any insurance plan. |
| 7 | 0.404 | counts_of_ICD10CM-cat_B97_1_or_more | Patient had 1 or more occurrences of the diagnosis: VIRAL AGENTS CAUSE DISEASES CLASSIFIED ELSEWHERE. |
| 8 | -0.396 | counts_of_CPT_94375_3_or_more | Patient had 3 or more occurrences of the procedure: Respiratory flow volume loop. |
| 9 | 0.377 | region_WEST | Patients residing in the WEST region. |
| 10 | 0.300 | counts_of_GPI8_43102010_3_or_more | Patient had been prescribed BENZONATATE 3 or more times. |
| 11 | -0.293 | counts_of_CPT_84520_1_or_more | Patient had 1 or more occurrences of the procedure: Urea nitrogen; quantitative. |
| 12 | 0.292 | counts_of_CPT_99213_3_or_more | Patient had 3 or more occurrences of the procedure: Office or other outpatient visit for the evaluation and management of an established patient, which requires at least 2 of these 3 key components: An expanded problem focused history; An expanded problem focused examination; Medical decision making of low complexity. Counseling and coordination of care with other physicians, other qualified health care professionals, or agencies are provided consistent with the nature of the problem(s) and the patient's and/or family's needs. Usually, the presenting problem(s) are of low to moderate severity. Typically, 15 minutes are spent face-to-face with the patient and/or family.. |
| 13 | 0.284 | counts_of_CPT_90656_1_or_more | Patient had 1 or more occurrences of the procedure: Influenza virus vaccine, trivalent (IIV3), split virus, preservative free, when administered to individuals 3 years and older, for intramuscular use. |
| 14 | 0.280 | counts_of_ICD10CM-cat_R05_2_or_more | Patient had 2 or more occurrences of the diagnosis: COUGH. |
| 15 | 0.262 | counts_of_CPT_99214_3_or_more | Patient had 3 or more occurrences of the procedure: Office or other outpatient visit for the evaluation and management of an established patient, which requires at least 2 of these 3 key components: A detailed history; A detailed examination; Medical decision making of moderate complexity. Counseling and/or coordination of care with other physicians, other qualified health care professionals, or agencies are provided consistent with the nature of the problem(s) and the patient's and/or family's needs. Usually, the presenting problem(s) are of moderate to high severity. Typically, 25 minutes are spent face-to-face with the patient and/or family.. |
| 16 | -0.261 | counts_of_CPT_71275_2_or_more | Patient had 2 or more occurrences of the procedure: Computed tomographic angiography, chest (noncoronary), with contrast material(s), including noncontrast images, if performed, and image postprocessing. |
| 17 | 0.261 | counts_of_ICD10CM-cat_J47_1_or_more | Patient had 1 or more occurrences of the diagnosis: BRONCHIECTASIS. |
| 18 | 0.260 | counts_of_GPI8_43102010_2_or_more | Patient had been prescribed BENZONATATE 2 or more times. |
| 19 | -0.255 | counts_of_ICD10CM-cat_K92_1_or_more | Patient had 1 or more occurrences of the diagnosis: OTHER DISEASES OF DIGESTIVE SYSTEM. |
| 20 | -0.255 | counts_of_CPT_99211_2_or_more | Patient had 2 or more occurrences of the procedure: Office or other outpatient visit for the evaluation and management of an established patient, that may not require the presence of a physician or other qualified health care professional. Usually, the presenting problem(s) are minimal. Typically, 5 minutes are spent performing or supervising these services.. |
| 21 | -0.251 | counts_of_HCPCS_J1100_1_or_more | Patient had 1 or more occurrences of the procedure: Injection, dexamethasone sodium phosphate, 1 mg. |
| 22 | -0.248 | age_18-39 | Patients between the ages of 18 and 39. |
| 23 | 0.241 | counts_of_ICD10CM-cat_T45_3_or_more | Patient had 3 or more occurrences of the diagnosis: PSN ADVRS EFF UNDRDOS PRIM SYS HEMATOLOG AGT NEC. |
| 24 | 0.235 | counts_of_CPT_88342_2_or_more | Patient had 2 or more occurrences of the procedure: Immunohistochemistry or immunocytochemistry, per specimen; initial single antibody stain procedure. |
| 25 | -0.232 | counts_of_CPT_80307_2_or_more | Patient had 2 or more occurrences of the procedure: Drug test(s), presumptive, any number of drug classes, any number of devices or procedures; by instrument chemistry analyzers (eg, utilizing immunoassay [eg, EIA, ELISA, EMIT, FPIA, IA, KIMS, RIA]), chromatography (eg, GC, HPLC), and mass spectrometry either with or without chromatography, (eg, DART, DESI, GC-MS, GC-MS/MS, LC-MS, LC-MS/MS, LDTD, MALDI, TOF) includes sample validation when performed, per date of service. |
| 26 | -0.230 | counts_of_CPT_36415_1_or_more | Patient had 1 or more occurrences of the procedure: Collection of venous blood by venipuncture. |
| 27 | -0.223 | ins_coverage_invalid/missing/unknown/other | Patient who has either an invalid, missing, unknown or miscellaneous insurance plan. |
| 28 | 0.220 | counts_of_GPI8_44201010_1_or_more | Patient had been prescribed ALBUTEROL 1 or more times. |
| 29 | -0.218 | counts_of_CPT_93000_3_or_more | Patient had 3 or more occurrences of the procedure: Electrocardiogram, routine ECG with at least 12 leads; with interpretation and report. |
| 30 | -0.215 | counts_of_ICD10CM-cat_M12_2_or_more | Patient had 2 or more occurrences of the diagnosis: OTHER AND UNSPECIFIED ARTHROPATHY. |
| 31 | 0.208 | counts_of_GPI8_04000020_3_or_more | Patient had been prescribed DOXYCYCLINE 3 or more times. |
| 32 | 0.205 | counts_of_GPI8_43995202_3_or_more | Patient had been prescribed HYDROCODONE POLISTIREX-CHLORPHENIRAMINE POLISTIREX 3 or more times. |
| 33 | -0.200 | counts_of_GPI8_57100040_1_or_more | Patient had been prescribed DIAZEPAM 1 or more times. |
| 34 | -0.199 | counts_of_prvspec_06_1_or_more | Patient had visited a Neurosurgery specialist 1 or more times. |
| 35 | 0.199 | counts_of_HCPCS_G0378_1_or_more | Patient had 1 or more occurrences of the procedure: Hospital observation service, per hour. |
| 36 | 0.198 | counts_of_GPI8_42200032_1_or_more | Patient had been prescribed FLUTICASONE 1 or more times. |
| 37 | -0.193 | region_SOUTH | Patients residing in the SOUTH region. |
| 38 | 0.193 | counts_of_ICD10CM-cat_F51_1_or_more | Patient had 1 or more occurrences of the diagnosis: SLEEP D/O NOT DUE SUBSTANC/KNOWN PHYSIOLOGL COND. |
| 39 | 0.192 | counts_of_GPI8_43102010_1_or_more | Patient had been prescribed BENZONATATE 1 or more times. |
| 40 | 0.187 | counts_of_CPT_31575_1_or_more | Patient had 1 or more occurrences of the procedure: Laryngoscopy, flexible fiberoptic; diagnostic. |
| 41 | 0.186 | counts_of_GPI8_46992005_1_or_more | Patient had been prescribed BISACODYL-PEG 3350-POT CHLORIDE-SOD BICARB-SOD CHLORIDE 1 or more times. |
| 42 | 0.184 | ins_coverage_commercial | Patient covered by a commercial insurance plan. |
| 43 | -0.184 | counts_of_ICD10CM-cat_I49_3_or_more | Patient had 3 or more occurrences of the diagnosis: OTHER CARDIAC ARRHYTHMIAS. |
| 44 | 0.179 | counts_of_prvspec_31_1_or_more | Patient had visited a Rheumatology specialist 1 or more times. |
| 45 | -0.178 | counts_of_CPT_99282_1_or_more | Patient had 1 or more occurrences of the procedure: Emergency department visit for the evaluation and management of a patient, which requires these 3 key components: An expanded problem focused history; An expanded problem focused examination; and Medical decision making of low complexity. Counseling and/or coordination of care with other physicians, other qualified health care professionals, or agencies are provided consistent with the nature of the problem(s) and the patient's and/or family's needs. Usually, the presenting problem(s) are of low to moderate severity.. |
| 46 | -0.178 | counts_of_HCPCS_J1100_2_or_more | Patient had 2 or more occurrences of the procedure: Injection, dexamethasone sodium phosphate, 1 mg. |
| 47 | 0.178 | counts_of_prvspec_35_1_or_more | Patient had visited a Vascular Surgery specialist 1 or more times. |
| 48 | -0.175 | counts_of_CPT_97162_1_or_more | Patient had 1 or more occurrences of the procedure: Physical therapy evaluation: moderate complexity, requiring these components: A history of present problem with 1-2 personal factors and/or comorbidities that impact the plan of care; An examination of body systems using standardized tests and measures in. |
| 49 | 0.175 | counts_of_HCPCS_G0439_1_or_more | Patient had 1 or more occurrences of the procedure: Annual wellness visit, includes a personalized prevention plan of service (PPS), subsequent visit. |
| 50 | -0.174 | region_OTHER/UNKN | Patients residing in a region unknown or unspecific to the known categorizations. |
